# Neural stem cells: are they the hope of a better life for patients with fetal-onset hydrocephalus?

**DOI:** 10.1186/2045-8118-11-7

**Published:** 2014-03-31

**Authors:** Montserrat Guerra

**Affiliations:** 1 Instituto de Anatomía, Histología y Patología, Facultad de Medicina, Universidad Austral de Chile, Valdivia, Chile

**Keywords:** Fetal-onset hydrocephalus, Neural stem cells, Regenerative therapy

## Abstract

I was honored to be awarded the Casey Holter Essay Prize in 2013 by the Society for Research into Hydrocephalus and Spina Bifida. The purpose of the prize is to encourage original thinking in a way to improve the care of individuals with spina bifida and hydrocephalus. Having kept this purpose in mind, I have chosen the title: *Neural stem cells, are they the hope of a better life for patients with fetal-onset hydrocephalus?* The aim is to review and discuss some of the most recent and relevant findings regarding mechanisms leading to both hydrocephalus and abnormal neuro/gliogenesis. By looking at these outcome studies, it is hoped that we will recognize the potential use of neural stem cells in the treatment of hydrocephalus, and so prevent the disease or diminish/repair the associated brain damage.

## Review

### Background

Fetal-onset hydrocephalus is one of the most challenging pediatric diseases. It has a variety of causes including the loss of cerebral tissue (cerebral atrophy), the excessive production of cerebrospinal fluid (CSF), or the obstruction of CSF pathways due to abnormal neuro- and gliogenesis [1]. Surgical treatment, such as CSF shunting and endoscopic third ventriculostomy (ETV) currently used for the treatment of children with fetal-onset hydrocephalus, is insufficient. An estimated 50% of shunts fail within two years and 20-50% of ETVs close up within five years; infections are also frequent [2]. Additionally, we do not yet know the consequences that may occur when CSF proteins are shunted into a confined space like the peritoneum that hosts a large number of immune system cells. There is a high possibility that shunt surgery and its sequelae generate auto-antibodies against specific CSF proteins. If these antibodies, or cells that produce them, eventually enter the brain, they may alter the neuronal physiology and exacerbate neurological deficits. Concerning ETV, we do not know the consequences of creating an opening through the membranous floor of the third ventricle to divert CSF into the subarachnoid space. Considering that the floor of the third ventricle is a highly specialized region for the secretion of regulatory factors affecting pituitary activity, ETV may possibly produce adverse effects on neuroendocrine regulation. It may also divert signaling molecules in the CSF away from their intended targets.

Several relatively recent publications have highlighted the importance and availability of appropriate secreted proteins (e.g. sonic hedgehog, insulin growth factor) and non-proteins (e.g. retinoic acid) distributed in the CSF, and their roles in development and maintenance of brain health [3-7]. Changes in CSF composition have a profound influence on the development and function of the brain. So, hydrocephalus, and the treatments available (shunting, ETV, and cauterizing the choroid plexus) may limit the availability of these positive factors for the developing and adult brain, resulting in severe life-long neurological deficits.

The financial and emotional costs of treatments to patients and their families are high and additional therapies are necessary. From where should solutions come? Considering the advances in medical science, these solutions will likely come from the more complete knowledge of the cellular and molecular processes that lead to the development of the disease [8]. They should come from laboratories where research generates information to be used in clinical practice. Thus, collaboration between the experimental and clinical investigators is fundamental and necessary to advance the search for new treatments.

A question emerges: What have we learned about fetal-onset hydrocephalus from laboratories? This review aims to show and discuss some of the most relevant recent findings regarding the mechanisms leading to both hydrocephalus and abnormal neuro/gliogenesis. It is focused on congenital hydrocephalus attributed to an obstruction in CSF pathways as consequence of abnormal neural stem/radial glial cell biology.

### Fetal-onset hydrocephalus is a pathology of neural stem cells

**Figure 1:**
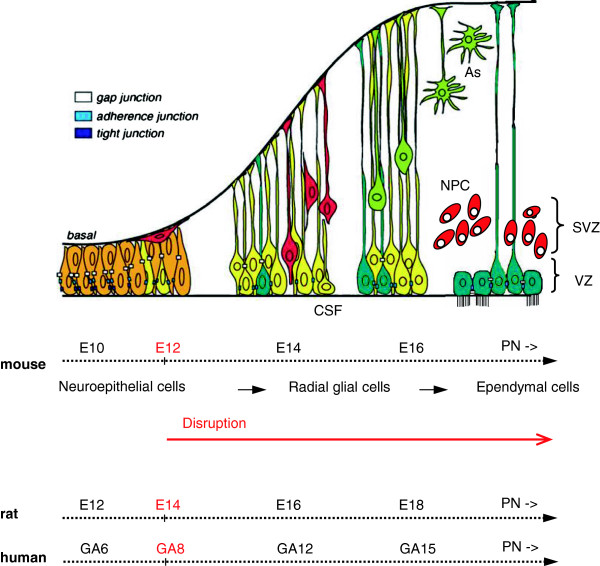
**A representation of normal cortical development showing the timed disruption of the ventricular zone in mouse (red arrow).** The phenotype of cells located in the ventricular zone (VZ) changes during normal brain development. Cell-cell junction pathology in the radial glial/neural stem cells and ependyma leads to the disruption of the VZ. In mouse, the exposure of the neural progenitor cells (NPC) localized in the subventricular zone (SVZ) occurs from embryonic day (E) 12.5 onward, when neurogenesis has been initiated. Note that the VZ cells contact the cerebrospinal fluid (CSF) during embryonic development. A timeline comparing neurogenesis events in the cortex of the mouse and rat in embryonic days (E) and in human with gestational age in weeks (GA) has been drawn, using a statistical model developed for Clancy *et al.*[68,69]. PN, postnatal; As, astrocytes.

It is now accepted that fetal-onset hydrocephalus is more than a disorder of CSF dynamics. It is also a brain disease. Recent studies have shown that hydrocephalus and abnormal neurogenesis, observed in both mutant animals and human hydrocephalic fetuses, share a common history: a pathology of the neural stem cells (NSC)/radial glial cells which are located in the ventricular zone (VZ) during embryonic development [9-12]. In the non-hydrocephalic state, these cells are joined by adherens and gap junctions (Figure 1). In hydrocephalus, cell-cell junction proteins accumulate abnormally in the cytoplasm of NSCs and ependymal cells and depending on the brain developmental stage it lead to their detachment from the VZ [10-12]. Disruption of the VZ may also be caused either by infections or intracerebral haemorrhage [13]. Only in a minority of cases is it associated with Mendelian inheritance, with X-linked hydrocephalus as the most common type [14,15].

**Figure 2:**
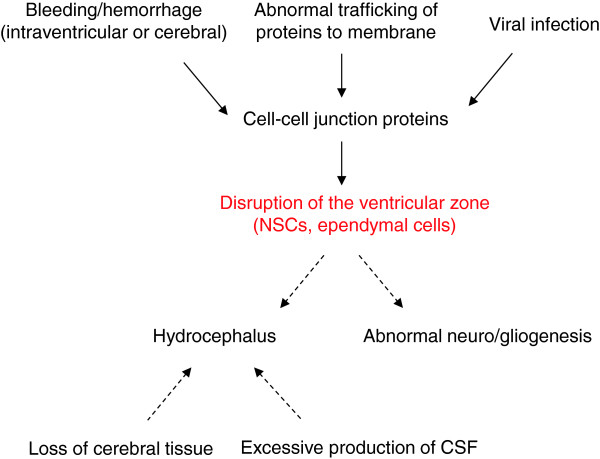
**Clinical outcome is related to the disruption of the ventricular zone.** The disruption of the ventricular zone (VZ) through effects on cell junction proteins can result from a variety of causes including abnormal trafficking of proteins to membrane, bleeding/hemorrhage and viral infections. The disruption of the ventricular zone in the telencephalon leads to abnormal neurogenesis. The disruption of the ventricular zone in the Sylvian aqueduct leads to aqueduct obstruction and hydrocephalus. Hydrocephalus may also result from loss of cerebral tissue (cerebral atrophy) and excessive production of cerebrospinal fluid (CSF).

In hydrocephalus disruption of the VZ is orderly and programmed. The disruption process starts early in the embryonic life and finishes during the first postnatal weeks (Figure 1, red arrow). In the mutant hyh mice the VZ disruption follows a caudo-rostral specific spatiotemporal pattern [16-18]. At the end of development certain regions are denuded of ependymal and subependymal cells, and other areas are not. The latter correspond to brain areas in which cells are joined by tight junctions, such as the circumventricular organs (e.g. subcommissural organ, choroid plexus, etc.). Disruption is clearly related to the clinical outcome (Figure 2). Various studies have consistently shown VZ disruption in the walls of the cerebral aqueduct [16-19] and in the pallium and ganglionic eminences of the telencephalon [10-12,20,21].

### What are the consequences of the disruption of the VZ?

Disruption of the VZ in the walls of the Sylvian aqueduct (SA) leads to SA stenosis during the prenatal life through fusion of the neuropil and results in SA obliteration prior to birth [16-19] (Figure 2). This finding is significant since it explains the addition of intraventricular obstruction after birth to what appeared (by imaging) to be communicating hydrocephalus pre-birth because the VZ disruption is only completed post-birth with the expected consequences [19].

The disruption of the VZ in the pallium and the ganglionic eminences leads to disturbances in neuro/gliogenesis, such as the displacement of neural progenitors (NPC) towards the CSF and the abnormal migration of neuroblasts in the cerebral cortex (Figure 2). This results in periventricular heterotopias (PH) that disrupt the normal organization and function of the cerebral cortex [10-12,20,21]. Since these cerebral malformations are present at birth, it may explain why a large number of hydrocephalic children develop neurological disorders (sensory, cognitive sequels, epilepsy) that are not resolved by CSF shunting [2].

PH represents a heterogeneous group of migrational disorders, characterized by nodules that are composed of neurons positioned ectopically along the lateral ventricular walls; they also behave as epileptogenic foci [22]. Prior studies have demonstrated an X-linked form of PH caused by mutations in the filamin A gene [23,24]. Mutations in other proteins, like ARFGEF2, CHS1, FAT4, coding for proteins involved in neuronal migration also lead to PH [23-25]. PHs are also a recurrent, but sometimes inconsistent, finding in terminal deletions of chromosome 6q [26]. PH associated with hydrocephalus (PHH) has primarily been reported in sporadic cases [10-12,21,23]. Genetic and molecular studies suggest that PHH is an etiologically heterogeneous condition that can be caused by different genes. One of these is the NAPA gene, coding for SNAPs (soluble NSF-attachment proteins) involved in the trafficking of proteins to the apical membrane [21,23,27].

It is estimated that epilepsy affects 6 to 30% of hydrocephalic patients [28,29]. However, no correlations have been found between the number of shunt revisions or the site of shunt placement and the risk of developing seizures [30,31]. It seems the most likely explanation for the development of seizure disorder in hydrocephalic patients is the presence of associated malformations (PH?) in the cerebral cortex. Evidence indicating that PH may result from radial glial/NSCs fiber disruption during embryonic development has been reported [10-12,21,32].

### Is stem cell therapy an alternative treatment for children with fetal-onset hydrocephalus?

Because disruption of the VZ is genetically determined and its consequences are widespread, the questions that arise are: is it possible to reverse this process and in doing so, will it have therapeutic benefits? The available evidence provides a ray of hope that it is possible to develop regenerative therapies based on the use of NSCs. These cells have two basic characteristics: they are self-sustaining and pluripotent [33,34]. This means that they proliferate for self-renewal; they also have the potential to differentiate into several cell types within the brain.

There are at least five advantages of using NSCs for nervous system repair strategies: NSCs are available in the embryonic and adult brain; they can be transplanted; and they migrate, differentiate and integrate into damaged areas. Furthermore, studies have shown that the ability of NSCs to migrate and differentiate into the required cell type depends on the damaged areas which release specific chemotactic factors [33,34]. Regenerative therapies are being used in the treatment of various neural disorders such as Parkinson’s and Alzheimer’s disease and multiple sclerosis [35-37] (Table 1).

**Table 1:** Clinical use of stem cells in the nervous system

**Pathology**	**References**
Alzheimer’s Disease	Abdel-Zalam *et al*., 2011 [87]
Parkinson’s Disease	Bjugstad *et al.*, 2008 [88]
Huntington’s Disease	Mc Bride *et al*., 2004 [89]
Multiple sclerosis	Pluchino *et al*., 2003 [90]; Martini *et al*., 2010 [54]; Rivera and Aigner 2012 [56]
Spinal cord injury	Obermair *et al.,* 2008 [91]
Brain stroke	Kelly *et al.,* 2004 [92]
Cerebral palsy	Cheng *et al*., 2013 [55]
Spina bifida aperta	Fauza et al., [77]; Li *et al.,* 2012 [57]

According to these studies and the revealed characteristics of NSCs a hopeful light is emerging for the future, so healthy NSCs can be transplanted into CSF to replace radial glial cells, neural progenitors and neuroblasts that are lost during the hydrocephalic process. This regenerative therapy might well repair the VZ and/or reverse the effects of the VZ disruption. Thus, malformations of the cerebral cortex that are found in the hydrocephalic children, which until now have been considered incurable, could have a realistic and promising alternative treatment. Such research could also open new ways to identify genes and epigenetic factors (growth factors, hormones) involved in neuronal and glial phenotypic differentiation allowing the design of new drugs to regulate the normal development of the cerebral cortex.

### Stem cell therapy for hydrocephalic children: is it science fiction or fact?

The development of regenerative therapies based on the use of NSCs to treat children with hydrocephalus is advancing well. Research led by Dr. EM. Rodríguez in Valdivia, Chile, using HTx hydrocephalic rats that receive transplants with NSCs, today represents the most advanced and promising alternative treatment for children with fetal-onset hydrocephalus. Preliminary studies have demonstrated that NSCs transplanted into CSF of hydrocephalic HTx rats at postnatal day 1 migrate to the disrupted areas and are integrated into nervous tissue [Rodríguez *et al.*, unpublished work]. The effect of this grafting on neuro/gliogenesis is under current investigation. The optimal timing for potential NSC transplantation into human CSF is unknown at this time. With the different causes leading to hydrocephalus in mind, a proposed stem cell therapy would have to be adapted to the different clinical backgrounds.

**Figure 3:**
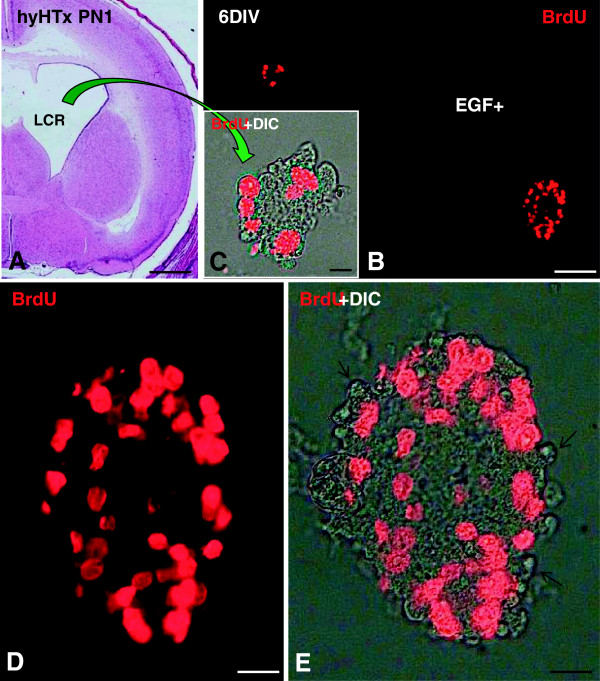
**Neurospheres can be grown from the cerebrospinal fluid of hydrocephalic HTx rats.** The disruption of the ventricular zone (VZ) leads to the abnormal translocation of neural stem cells (NSC) and neural progenitor cells (NPC) into the ventricle [10,11]. **A**. Coronal section of hydrocephalic brain stained with haematoxylin-eosin. Cells collected from the cerebrospinal fluid (CSF) of hydrocephalic HTx rats at postnatal day 1 (PN1) were cultured in medium containing 20 ng/ml epidermal growth factor (EGF) for six days. BrdU was added to the culture medium for the last 24 h. **B-D**. Immunofluorescence of large and small neurospheres with anti-BrdU stain showing proliferative (NSC/NPC) cells. **C**. High magnification of a small neurosphere. **D**. High magnification of a large neurosphere. **E**. After 6 days *in vitro* neurospheres had an irregular outline, with cells “disrupting” from its periphery (arrows) resembling that of the VZ of the living mutant. DIC, Differential interference contrast microscopy. Scale bars: A 600 μm; B 50 μm; C 5 μm; D, E 10 μm.

Investigations in Chile have already borne fruit. NSCs collected from the CSF of hydrocephalic mutant rats and hydrocephalic human fetuses proliferate to form neurospheres (Figure 3). NSCs forming these neurospheres express the same cell junctional pathology as NSCs of pallium (Figure 3) [Rodríguez *et al.*, unpublished work]. These accomplishments open the door for the use of neurospheres for diagnostic purposes, such as brain biopsies to investigate cell and molecular alterations underlying the disease. In this context, it is significant to note that although nearly 40% of hydrocephalus patients have a possible genetic cause, to date only the L1-CAM gene has been identified in humans [15].

It is worth noting that although sphere formation has been extensively utilized by research groups as an assay for stem cells to isolate, maintain, and expand NSCs [38], like all technologies, it is not without limitations, some of which have been extensively reviewed by different authors [39-41]. Furthermore, the growth and differentiation of neurospheres depend on cell culture density, the clonality of spheres and the presence of exogenous growth factors such as epidermal growth factor (EGF) or fibroblast growth factor (FGF) [39-41]. While new assays and markers for stem cells and their progeny have yet to be developed to overcome some of the limitations of the neurospheres assay, these have to be kept in mind before extrapolating results or translating the experimental transplantation of neurosphere-derived cells to the clinical setting.

### What will be the source of NSCs to be transplanted?

Today it is well known that NSCs are present in the embryonic and adult brain. During embryonic development, the NSCs are in the VZ whereas during adulthood there are “neurogenic niches” where specific postnatal neurogenesis persists. Postnatal neurogenesis in these areas is associated with the renewal of the olfactory epithelium, memory formation and cerebral metabolic homeostasis [42].

#### Human embryonic stem cells (hES cells)

These pluripotent cells have a major clinical potential for tissue repair, with their proponents believing that they represent the future relief or cure for a wide range of common disabilities [43,44]. It is suggested that the replacement of defective cells in a patient by transplantation of hES cell‒derived equivalents would restore normal function. Recently they have been used to generate choroid plexus (CP) cells [45]. Because of the essential developmental and homeostatic roles of CP cells relating to the CSF and the resulting blood-CSF barrier, the transplant of hES cells into CSF and differentiation into CP cells could represent a new approach for developing a therapy for hydrocephalus. Interestingly, CP cells have been grafted into the lateral ventricle of normal and hydrocephalic HTx rat littermates [Rodríguez *et al.,* unpublished work]. One week after transplantation the transplanted CP retained the cellular and molecular characteristics of living CP such as the expression of transthyretin in the cytoplasm and aquaporin1 at the apical plasma membrane. Since the grafted CP cells did not become re-vascularized, they would not secrete CSF but could be an extra source of trophic factors.

Despite the potential benefit of using hES cells in the treatment of disease, their use remains controversial because of their derivation from human pre‒implantation embryos [46]. The most controversial variant of this is the transfer of a somatic cell‒nucleus from a patient to an enucleated oocyte (unfertilized egg) in order to produce hES cells genetically identical to that patient for ‘autologous’ transplantation (so‒called ‘therapeutic’ cloning) which avoids tissue rejection [47,48]. hES cells are currently discussed not only by the biologists by whom they were discovered but also by the medical profession, media, ethicists, governments and politicians. The question ‘Can these cells be isolated and used and, if so, under what conditions and restrictions?’ is presently high on the political and ethical agenda, with policies and legislation being formulated in many countries to regulate their derivation.

#### Mesenchymal stem cells (MSCs)

Fortunately, the use of embryos is not the only and best way to obtain stem cells. Populations of stem cells reside within different tissues, representing an alternative source of cells that can be harvested at low cost, isolated with minimal invasiveness, and without ethical objections, are emerging as a replacement for hES cells [49]. In this context, various studies have shown the presence of a large MSCs population in umbilical cord blood, placental membranes and amniotic fluid [50]. In human and veterinary research, stem cells derived from these tissues are promising candidates for disease treatment, specifically for their plasticity, their reduced immunogenicity, and high anti-inflammatory potential [51-53]. Increasing research of molecular mechanisms that drive the differentiation of MSCs to NSCs [54,55] enhance the likelihood that MSCs could be useful in the treatment of neurological diseases. In fact, already transplantation of MSCs promotes myelin repair and functional recovery in different animal models of multiple sclerosis [56]. Transplantation also confers beneficial effects when MSCs are transplanted *in utero* into rat fetuses with spina bifida [57].

#### Induced pluripotent stem cells (iPSCs)

Today, thanks to progress in the ability to manipulate cell identity, it is possible to reprogram adult skin fibroblasts into induced pluripotent stem cells (iPSCs). They represent a new tool for drug discovery, disease modeling and a new hope for stem cell therapies [58-62]. In a recent paper Lancaster *et al.*[63] described a method for growing three-dimensional neural tissue from human PSCs and used it to model microcephaly. The resulting cerebral organoids reached up to 4 mm in size and contained polarized radial glia-like stem cells that surround a fluid-filled cavity resembling the lateral ventricle in the developing brain. This model may serve as a valuable *in vitro* platform for studying the molecular mechanisms that regulate the development of brain cortex and could provide an innovative and complementary approach for the study of the VZ disruption leading to hydrocephalus and abnormal neuro/gliogenesis *in vitro*.

### When should NSC transplantation be performed?

The hyh mutant mouse develops long-lasting hydrocephalus and is a good model for investigating neuropathologic events associated with hydrocephalus. The study of brains using lectin-binding, bromodeoxyuridine labeling, immunochemistry, and scanning electron microscopy has revealed that certain events related to hydrocephalus follow a well-defined pattern [16-18]. A program of VZ disruption is initiated at embryonic day (E)12.5 at the cerebral aqueduct and terminates at the end of the second postnatal week at the telencephalon. After the third postnatal week the disrupted areas remain permanently devoid of ependyma. The etiopathogenesis of hydrocephalus in the HTx rat is different from that in the hyh mouse. A distinct malformation of the subcommissural organ (SCO) is present as early as E15 leading to stenosis of the cerebral aqueduct at E18 and dilation of the lateral ventricles starts at E19 [64,65]. At this stage, the VZ disruption is initiated in the pallium of telencephalon and terminates at the end of the first postnatal week.

In hydrocephalic human fetuses the VZ cells, either NSCs or ependymal cells, undergo disruption [10-12,19,20]. In young hydrocephalic fetuses (21, 22 weeks of gestational age, GA) disruption occurs in the pallium while in 40-week GA fetuses the disruption extended to other regions of the lateral ventricles. The VZ disruption in the pallium was found to be related to the presence of PHs in the telencephalon and the abnormal displacement of the NSCs into the ventricle [10-12,20]. In full-term hydrocephalic fetuses with spina bifida aperta (37-, 39-and 40-week GA), both early and late stages of the VZ disruption were concurrently present in the cerebral aqueduct [19] indicating that at these stages, the VZ disruption is still an ongoing process. In these fetuses it may be suggested that such a process would have continued after birth. Only when this program is completed, the obliteration of SA occurs, triggering a severe hydrocephalus [19].

**Figure 4:**
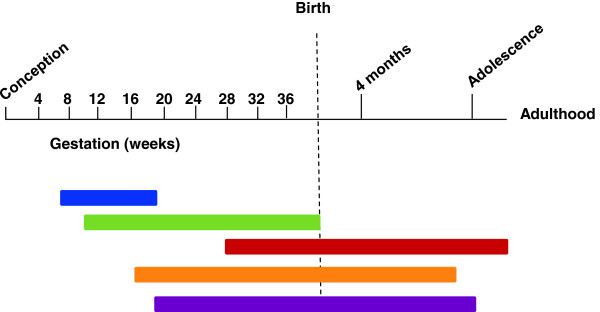
**Timeline of major events in the human brain development from conception through to adulthood.** Blue bar, neuronal proliferation; Green bar, neural migration; Red bar, myelination; Orange bar, synaptogenesis; Purple bar, apoptosis (adapted from [93]).

Human brain development is a protracted process that begins in the third gestational week [66,67]. Neuron proliferation begins in the sixth gestational week and is largely complete by mid-gestation. As they are produced, neurons migrate to different brain areas where they begin to make connections with other neurons establishing rudimentary neural networks. Although at the end of the prenatal period major fiber pathways, including the thalamocortical pathway, are complete, brain development continues for an extended period postnatally (Figure 4).

So far, the information obtained from mutant animals and human hydrocephalic fetuses strongly supports the idea that pallial disruption occurs while cortical neurogenesis is proceeding (Figure 4). Ideally, transplants should be done when the VZ disruption occurs. In this context, NSC transplantation should occur in the early stages of fetal development during the process of neuron production in the cortex [66-69]. There are reasons to be optimistic. Recent progress made with fetal surgery *in utero*[70,71] allows us to foresee that these surgeries might become increasingly safe and that transplantation of NSCs *in utero* might become feasible. The main priority must be maternal and fetal safety and avoiding preterm labor while achieving the aims of the surgery [72]. Open fetal surgery is possible between approximately 18 and 30 weeks of gestation: the limitations being fetal size and fragility before 18 weeks and, and increased risk of premature labor after 30 weeks [72]. Clearly it would be preferable to deliver the child and perform surgery *ex utero* instead. Prenatal repair of neural tube defects such as myelomeningocele and spina bifida is an increasing option in the United States. Although the procedure is technically challenging, children treated with open fetal repair have significantly improved outcomes compared to children whose defects are repaired shortly after birth [73,74]. Specifically, fetal repair reduces the rate of ventriculoperitoneal shunt dependence for hydrocephalus and improves motor skills at 30 months of age compared to those with post-natal repair. As a result, open fetal repair of spina bifida is now considered standard of care at specialist centers. Most children born with spinal bifida also have hydrocephalus. Why not use open fetal surgery to repair the neural tube defects as an opportunity to transplant NSCs? Indeed, fetal cell therapies have been employed in the treatment of human congenital hematological diseases and immunodeficiency [75,76], and in experimental animal models of spina bifida and myelomeningocele [57,77]. These studies have also shown that the safety of stem cell therapy depends on various factors including the differentiation status and proliferative capacity of the grafted cells, the route of administration and the long-term survival of the graft.

### What should we expect of repair?

It was first assumed that stem cells directly replace lost cells, but it is now becoming clearer that they might be able to protect the nervous system through mechanisms other than cell replacement, such as the modulation of the immune system [51-53,78,79]. Worthy of note is the remarkable capacity of NSCs and NPCs to cross-talk with endogenous cells and to remodel the injured nervous system when they are applied [80-83]. Further, a recent study demonstrated an astrocytic reaction in the disrupted VZ in hydrocephalus in that astrocytes acquire morphological and functional characteristics of ependymal cells, suggesting that they function as a CSF–brain barrier involved in water and solute transport [84]. Such remodeling would help to re-establish lost functions at the brain parenchyma–CSF interface. Therefore, we postulate that grafting of NSCs into hydrocephalic brain would result in the replacement of cells and/or the generation of a protective microenvironment to prevent the progressive disruption of the VZ and to enhance favorable glial responses.

## Conclusions

Today it is accepted that fetal-onset hydrocephalus is more than just an alteration in CSF dynamics. It is also a brain disorder. Children born with hydrocephalus also have a severe malformation of the cerebral cortex and cognitive deficits. These deficiencies are not successfully addressed by shunting or by ETV. The transplantation of NSCs is emerging as a great hope to correct brain maldevelopment, helping to reduce the brain damage and promoting regeneration and repair through direct cell replacement and neurotrophic and immunomodulatory mechanisms. Many questions regarding the application of stem cells remain unanswered, particularly tumorigenicity, immune rejection and danger of gene manipulation. However, transplantation is expected eventually to become as common a practice as other treatments for neurological diseases ( [85,86], Table 1). To achieve the NSCs transplantation goal for hydrocephalus/spina bifida will require better integration of experimental and clinical activities to reveal the genetic control of identity and growth of stem cells, identify factors that predispose differentiation into specific neuronal or glial lineages, and implement surgical techniques that allow safe NSCs transplants. Expectations, though guarded, are high. If both basic and clinical researchers join forces, the dream of providing a better life to children with hydrocephalus will draw closer with each passing day.

## Abbreviations

CSF: Cerebrospinal fluid; EGF: Epidermal growth factor; ES: Embryonic stem cells; ETV: Endoscopic third ventriculostomy; FGF: Fibroblastic growth factor; GA: Gestational age; iPSC: Induced pluripotent stem cells; MSC: Mesenchymal stem cells; NSC: Neural stem cells; NPC: Neural progenitors; PH: Periventricular heterotopias; SA: Sylvian aqueduct; SCO: Subcommissural organ; VZ: Ventricular zone.

## Competing interests

The author declares that she has no competing interest.
